# Still Valid: Clearance of *Pseudocapillaroides xenopi* from a *Xenopus laevis* Colony Using the 1997 Levamisole Immersion Protocol

**DOI:** 10.3390/ani16213338

**Published:** 2026-10-23

**Authors:** Maja Strunk, Ardalan Ahmadvand, Lisa Ernst, René H. Tolba, Leonie Tix

**Affiliations:** Institute for Laboratory Animal Science and Experimental Surgery, RWTH Aachen University, 52074 Aachen, Germanyltix@ukaachen.de (L.T.)

**Keywords:** *Xenopus laevis*, *Pseudocapillaroides xenopi*, cutaneous capillariasis, levamisole, colony health monitoring, skin scraping

## Abstract

*Pseudocapillaroides xenopi* is a capillariid nematode of the epidermis of *Xenopus* spp. and a cause of “flaky” or “gray skin disease” in laboratory colonies. The only published regimen for eradicating it from an entire colony, which is a levamisole immersion, dates from 1997. We report its unmodified application in a colony of approximately 480 adult female *X. laevis*. An index animal with pale, roughened skin and severe weight loss was diagnosed by PCR. Screening all 42 cohabitants of three tanks each holding one animal with macroscopic skin abnormalities gave a prevalence of 21.4% (9/42), with seven of them clinically inconspicuous. Two four-day levamisole baths (12 mg/L) ten days apart, with tank cleaning and dedicated equipment, were applied colony-wide. Post-treatment skin scrapings were negative in 42/42 animals, gross lesions resolved, body weight was maintained and no further cases have occurred. The 1997 protocol remains fully effective. As the untreated infection is ultimately fatal and the alternative to treatment is depopulation, a regimen that reliably clears a colony is also a refinement.

## Simple Summary

*Pseudocapillaroides xenopi* is a skin parasite that can cause severe disease, weight loss, and death in *Xenopus laevis* frogs. Although a levamisole immersion treatment was described almost 30 years ago, its effectiveness had not been reassessed under current laboratory conditions. We report an outbreak in a colony of approximately 480 adult female frogs and demonstrate that the original treatment protocol, combined with hygiene measures, successfully eliminated the parasite without observable adverse effects. Most infected animals showed no visible clinical signs, highlighting the importance of active screening of all tank inhabitants when a single affected animal is identified. Our findings confirm that this simple treatment strategy remains an effective and practical option for controlling *P. xenopi* infections while avoiding unnecessary euthanasia or depopulation of the affected colony.

## 1. Introduction

The South African clawed frog (*Xenopus laevis*) is an established model organism in developmental biology, genetics and reproductive physiology [1,2]. Amphibian skin contributes to respiration, osmoregulation and immune defense, so dermatological disease has consequences beyond the local lesion [3,4,5]. The epidermal capillariid nematode *Pseudocapillaroides xenopi* is specific for *X. laevis* and the western clawed frog (*Xenopus tropicalis*) and causes “flaky” or “gray skin disease” with depigmentation, a roughened skin surface, anorexia, cachexia, secondary bacterial infection and, in individual cases, death [2,6,7]. The life cycle is direct: eggs deposited in the epidermis are released with desquamated skin, so auto-infection and infection of cohabitants continue within a tank [8].

The only published protocol for eradicating *P. xenopi* from an entire colony is the levamisole immersion regimen of Iglauer et al. [8]. Almost three decades later, with levamisole resistance spreading in nematodes of veterinary importance [9], it is not self-evident that it still performs as described. We report an outbreak in a colony of approximately 480 *X. laevis* and show that the protocol, applied without modification, achieved parasitological clearance, resolution of lesions and stable body weights.

## 2. Materials and Methods

### 2.1. Colony

The colony comprises approximately 480 adult female *X. laevis* without in-house breeding, held in a semi-closed recirculating system consisting of 30 tanks arranged in parallel (L × W × H: 100 × 50 × 25 cm, ~75 L) (Aqua Schwarz, Göttingen, Germany) at 20 ± 1 °C in stable groups of 8–18 with refuges and once-daily feeding except on weekends. Water treatment is carried out using mechanical filtration systems, demineralization, salting, activated carbon filtration, and UV irradiation. Filters and UV lamps are replaced at least twice a year. Within 24 h, 3–5% of the total water volume is replaced by fresh water through continuous flushing. Water quality is checked weekly using test strips (standard values: nitrate < 25 mg/L, nitrite < 0.5 mg/L, and pH 6.8–7.6). The day–night cycle is 12:12 h. No case of cutaneous capillariasis had been recorded since the facility opened in 2018.

### 2.2. Diagnostic Work-Up

**Figure 1:**
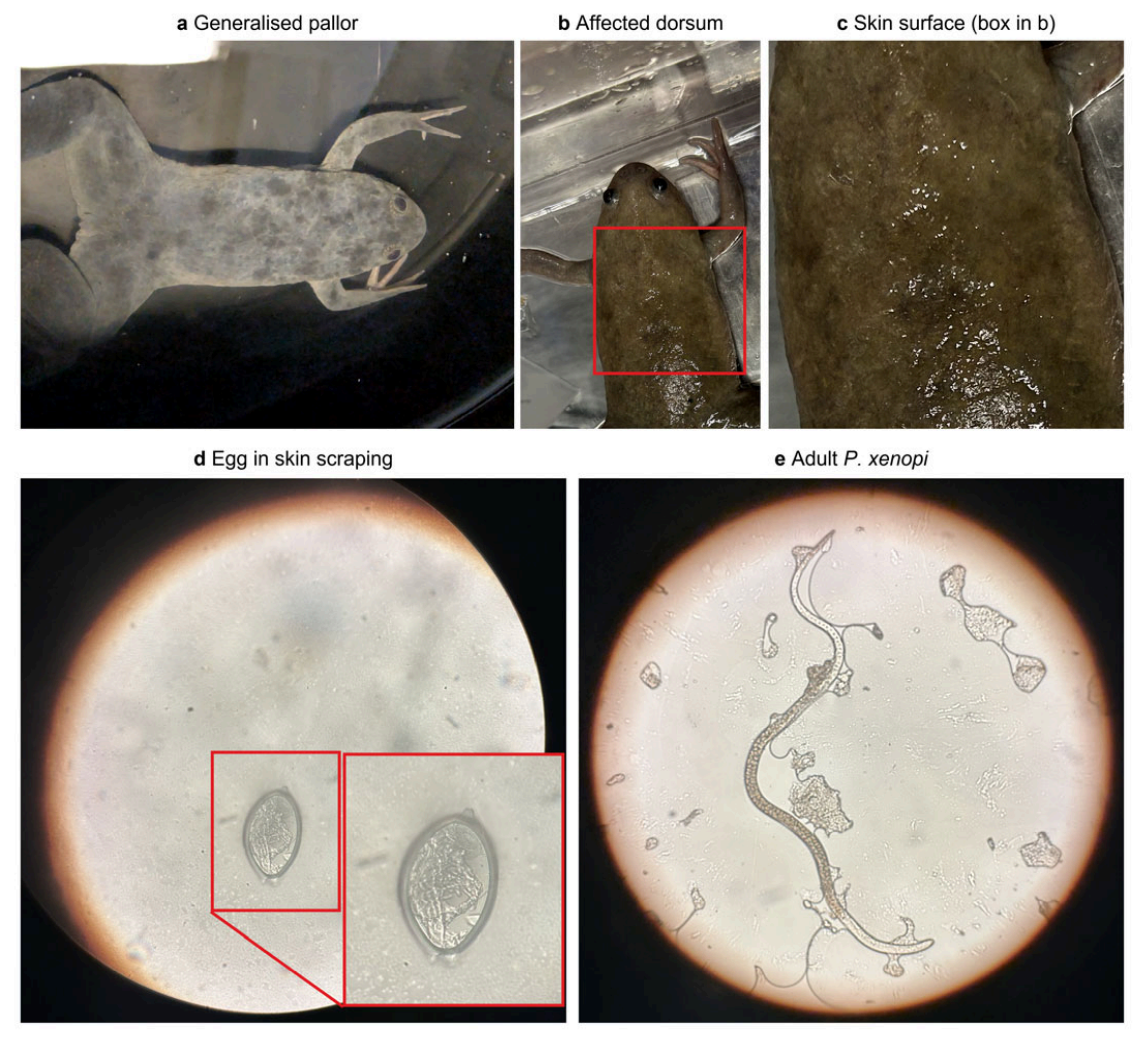
Cutaneous capillariasis in *Xenopus laevis*: gross lesions (**a**–**c**) and parasitological diagnosis (**d**,**e**). (**a**) Generalized pallor and depigmentation of the skin. (**b**) Dorsum of a second affected animal; red box, region enlarged in (**c**). (**c**) Roughened, focally pale skin surface with desquamated material. (**d**) Capillariid egg with bipolar plugs in an unstained skin scraping; inset, enlargement of the boxed region. (**e**) Adult *Pseudocapillaroides xenopi*. Unstained wet preparations; the original magnification was not recorded, so no scale bars are given.

Initially, two animals with generalized pale, depigmented and roughened skin and progressive weight loss were noticed (Figure 1a–c). Skin swabs incubated aerobically and anaerobically yielded no relevant bacterial or fungal growth, and the animals had to be euthanized for animal welfare reasons. The next animal was euthanized via decapitation under general anesthesia because it reached the humane endpoint defined by body condition score [1,10]. A skin scraping was positive for *P. xenopi* DNA by PCR in a *Xenopus*-specific diagnostic panel (GVG Diagnostics, Leipzig, Germany). As three more animals in three different tanks showed macroscopic skin abnormalities, skin scrapings were taken with cover slips from the back, neck and flank of all 42 animals in these three tanks and examined by light microscopy.

### 2.3. Treatment and Hygiene

Levamisole hydrochloride (CP-Pharma, Burgdorf, Germany) was used exactly as described by Iglauer et al.: immersion at 12 mg/L in static tanks (~60 mg per animal) for four consecutive days, repeated after ten days in normal system water. Feeding was unchanged and animals were inspected daily. All 30 tanks of the affected housing system were treated, including those without clinical cases. In asymptomatic tanks, the 3–5 lightest animals per tank were scraped as a spot check, being the most likely to be infested. Home tanks and refuges were drained, cleaned and flushed with hot water, equipment was tank-dedicated, gloves were changed between animals, and work proceeded from treated to not-yet-treated tanks. Because only three tanks could be processed simultaneously, colony-wide treatment extended over 3 months.

### 2.4. Follow-Up

Five days after the second bath, all 42 animals of the three index tanks were inspected, skin scraped and examined again. Eight animals with microscopically confirmed pre-treatment infection were weighed before and after treatment. Thereafter, the colony was monitored for six months through daily clinical inspection during routine care.

## 3. Results

Microscopy of the 42 index-tank animals demonstrated nematode eggs, live and dead adults and characteristic intraepidermal tunnels, confirming the PCR diagnosis (Figure 1d,e). Nine of 42 animals (21.4%) were positive. Of the three animals with gross lesions, two were positive, meaning seven of the nine positive animals were clinically inconspicuous. Spot checks in the remaining tanks were negative.

**Figure 2:**
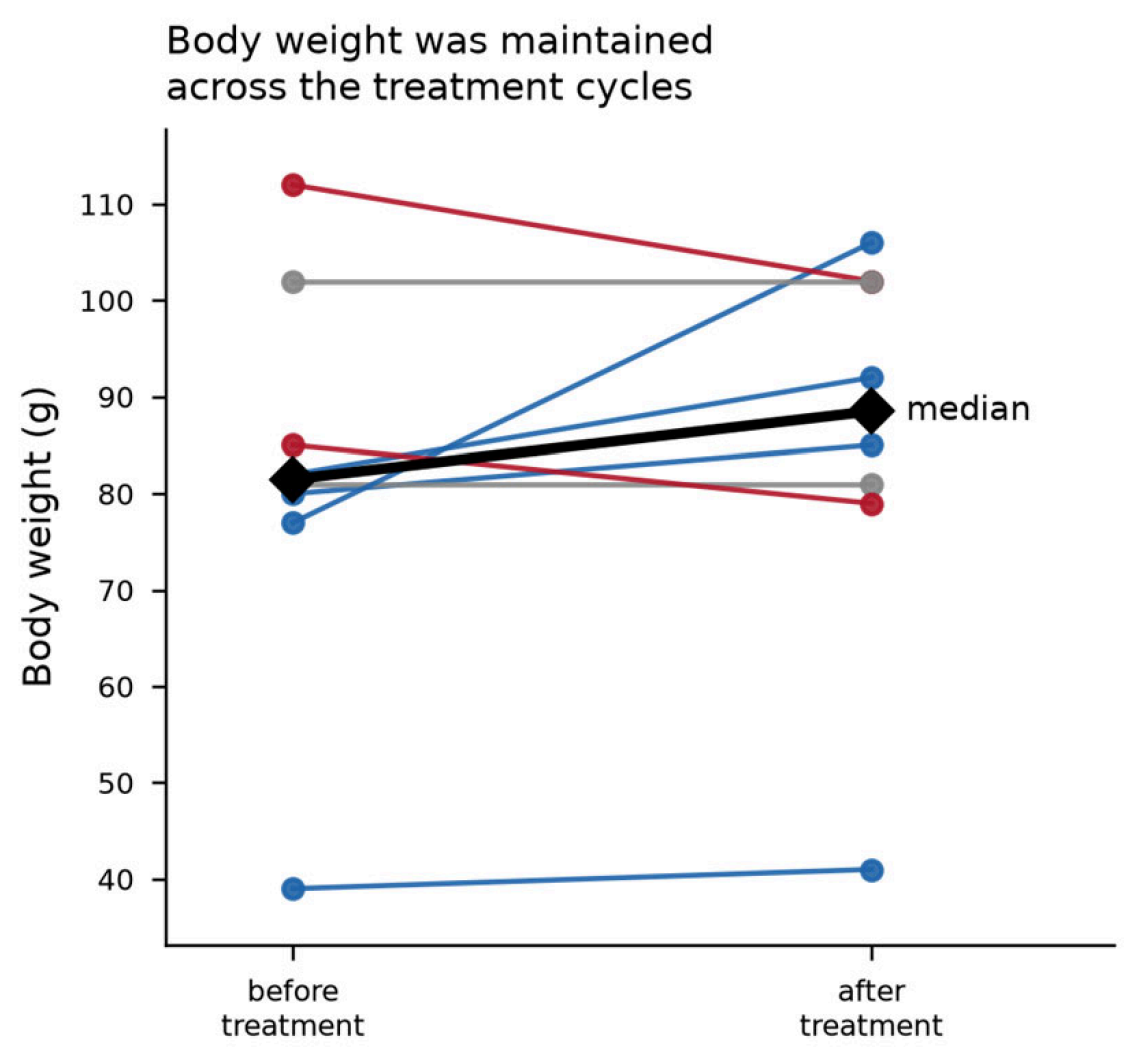
Body weight of eight animals with confirmed *Pseudocapillaroides xenopi* infection before and after two levamisole baths (12 mg/L, four days each, ten days apart). Blue = gain; red = loss; gray = unchanged.

The levamisole baths were tolerated without clinically observable adverse effects, and no animal died. After the second cycle, no parasitic stages were detected in skin scrapings from the 42 re-sampled animals and the gross skin changes had resolved. Median body weight of the eight followed animals was 81.5 g (range 39–112) before and 88.5 g (range 41–106) after treatment: four animals gained weight, two were unchanged and two lost a small amount, so weight was maintained rather than demonstrably restored in this small sample (Figure 2). No new clinical case has occurred in the following 6 months after treatment was completed (during which follow-up consisted of clinical observation only) and no animal had to be euthanized after the diagnosis was established, and ongoing experiments continued with scheduling adjustments only.

## 4. Discussion

The central finding is that the levamisole immersion regimen published by Iglauer et al. in 1997 [8] still eradicates *P. xenopi*. Applied without modification 29 years later, it produced complete absence of parasitic stages on follow-up microscopy, resolution of the macroscopic skin changes and no further cases colony-wide. Levamisole resistance is spreading among nematodes of veterinary importance [9]. To the best of our knowledge, no data have been published on levamisole resistance in nematodes parasitizing amphibians including *P. xenopi.* Vineer et al. reported levamisole resistance in nematodes of European livestock, with a prevalence of up to 48% depending on the species [11]. Against this background, the confirmation that levamisole successfully cleared the *P. xenopi* infection is not trivial and supports the regimen as a first-line option for *Xenopus* colonies. Because the drug was deliberately combined with hygiene measures, our data confirm the published package rather than levamisole alone. The second treatment cycle is an integral part of the regimen: levamisole, a nicotinic acetylcholine receptor agonist, causes spastic paralysis of motile nematode stages but does not act on eggs embedded in the epidermis [8,12]. The ten-day interval is intended to reach stages that have emerged after the first bath before they become fertile [8].

The second important finding of this case concerns the prevalence of infection among the screened animals and detection of affected animals. Each of the three affected tanks was identified from a single visibly abnormal animal, yet screening all cohabitants revealed a prevalence of 21.4%, most positive animals being clinically normal. Infected frogs can remain asymptomatic for long periods and decompensate under stress, when the burden escalates rapidly [2,13]. Gross inspection alone is therefore not a sufficient monitoring tool, and a single index case should trigger examination of every cohabitant, not only of lesion-bearing animals. Moreover, the gross lesions are non-specific; in our case, the differential diagnoses were bacterial dermatitis, septicemia, saprolegniasis, chytridiomycosis and water-quality-related dermatopathy, so parasitological confirmation is essential. Skin scraping proved to be a useful, rapid and inexpensive method for detecting infection; quantitative PCR on aquarium sediment offers a non-invasive alternative for large colonies [2].

The welfare significance of a regimen that still works is considerable. Untreated cutaneous capillariasis is progressive and ultimately fatal: animals develop anorexia and cachexia, are predisposed to secondary bacterial infection and may have to be euthanized, as our index animal was, since the epidermis is also a respiratory, osmoregulatory and immune organ [14]; the burden is not confined to the skin [15]. The immersion baths caused no observable distress and required no injection, anesthesia or restraint beyond routine handling, so the severity of the intervention was far below that of the disease. The alternative to an effective treatment is depopulation and re-derivation, which for ~500 animals would mean killing healthy, treatable individuals and repeating the experiments they were in.

The epidemiological route of introduction could neither be determined nor retrospectively traced: the outbreak followed the arrival of an externally purchased cohort, but the source colony reported no comparable infection and no transport material remained for sampling. The episode nevertheless argues for quarantine and an arrival examination including a skin scraping, consistent with FELASA–AALAS recommendations for aquatic facilities [16].

Several limitations apply to this case report due to its characteristics as an investigation of a disease case. There was no untreated control group, so the contribution of levamisole cannot be separated from that of the hygiene measures. Follow-up used a single scraping per animal, so a very low residual burden cannot be excluded formally, although negative spot checks and absence of new cases argue against it. Prevalence was determined in the three index tanks only, and the body-weight follow-up is descriptive. Finally, treated and untreated animals coexisted in one room for 3 months, which is defensible only with the directional workflow and tank-dedicated equipment described. The facility itself has also installed filtration systems and UV lights for disinfection, thereby reducing the spread of germs from this source as well. Filtration systems and UV lamps for disinfection, which had been installed even before the outbreak, also reduced the spread of germs from this source.

## 5. Conclusions

The levamisole immersion protocol of Iglauer et al. remains an effective, well-tolerated and practicable strategy for eradicating *P. xenopi* from *X. laevis* colonies when combined with tank cleaning and consistent prevention of mechanical carry-over. Because most infected animals were clinically normal, a single index case should prompt examination of all cohabitants. Confirming that the regimen still works has direct welfare value: a progressive, ultimately fatal disease is treated with a low-severity procedure, and depopulation of an affected colony becomes unnecessary.

## Data Availability

The data presented in this study are available from the corresponding author upon reasonable request.

## Abbreviations

The following abbreviations are used in this manuscript:

*X. laevis*	*Xenopus laevis*
*P. xenopi*	*Pseudocapillaroides xenopi*
DNA	Deoxyribonucleic acid
PCR	Polymerase Chain Reaction
FELASA	Federation of European Laboratory Animal Science Associations
AALAS	American Association for Laboratory Animal Science
